# Identification and expression pattern analysis of miRNAs in pectoral muscle during pigeon (*Columba livia*) development

**DOI:** 10.7717/peerj.11438

**Published:** 2021-06-23

**Authors:** Xun Wang, Peiqi Yan, Siyuan Feng, Yi Luo, Jiyuan Liang, Ling Zhao, Haifeng Liu, Qianzi Tang, Keren Long, Long Jin, Jideng Ma, Anan Jiang, Surong Shuai, Mingzhou Li

**Affiliations:** 1 Institute of Animal Genetics and Breeding, College of Animal Science and Technology, Sichuan Agricultural University, Chengdu, Sichuan, China; 2 College of Veterinary Medicine, Sichuan Agricultural University, Chengdu, Sichuan, China

**Keywords:** Development, miRNAs, Pigeon, Pectoral muscle, Small RNA-seq

## Abstract

MicroRNAs (miRNAs) are a group of crucial regulators in the process of animal growth and development. However, little is known about the expression and function of miRNAs in pigeon muscles. To identify the miRNAs participating in the rapid development of pigeon pectoral muscles and quantitate their expression levels of pectoral muscles in different age stages, we performed miRNA transcriptome analysis in pigeon pectoral muscles by sequencing small RNAs over three different age stages (1-day old, 28 days old, and 2 years old). Dual-luciferase reporter assay was applied to validate the interaction between miRNA and its target gene. We identified 304 known miRNAs, 201 conserved miRNAs, and 86 novel miRNAs in pigeon pectoral muscles. 189 differentially expressed (DE) miRNAs were screened out during pigeon development. A short time-series expression miner (STEM) analysis indicated 89 DE miRNAs were significantly clustered in a progressively decreasing expression profile, and mainly enriched in biosynthesis-related GO categories and signaling pathways for MAPK and TGF-*β*. Dual-luciferase reporter assay indicated that a progressively down-regulated miRNA (miR-20b-5p) could directly target Krüppel-like factor 3 (*KLF3*) gene. To sum-up, our data expand the repertoire of pigeon miRNAs and enhance understanding of the mechanisms underlying rapid development in squabs.

## Introduction

Pigeons (*Columba livia*) have been reared for meat production and racing since at least 2500 BC Sales & Janssens (2003). In China, pigeon is the fourth-largest domestic poultry in breeding scale following chicken, duck and goose. As the largest muscle in pigeons, pectoral muscles are important for flight ability and meat production, constituting approximately 15% of total body weight (James & Meek, 1976). The pectoral muscle is the dominant avian flight muscle producing mechanical work during the downstroke and pronation of the wing (Biewener, 2011). The pectoral muscle in pigeon squabs develops rapidly; Gao et al. (2016) reported the weight of the pectoral increased by a striking 173.3-fold from 1- to 35-day-old birds. To date, the underlying mechanism of such rapid growth in pigeon pectoral muscles remains poorly understood. Muscle development is a complicated process involving proliferation and differentiation of myogenic progenitor cells, and formation of fused myotubes and myofibers (Yang et al., 2016). In birds, the number of myofibers present in muscle is defined at hatching (Smith, 1963), and post-hatch skeletal muscle growth is dependent on the accumulation of nuclei in myofibers and hypertrophy of existing fibers (Li et al., 2012). More specifically, skeletal muscle growth is dictated by the fusion of proliferating satellite cells to myofibers, ultimately causing an increase in DNA content and protein synthetic capacity (Yin et al., 2014). This process is a highly regulated multi-step process orchestrated by myogenic regulatory factors including Myf5, Myf6, MyoD, and myogenin (Bryson-Richardson & Currie, 2008).

As a group of endogenous small non-coding RNAs, microRNAs (miRNAs) are crucial post-transcriptional regulators of gene expression that usually bind to the 3′-UTR of target mRNAs, leading to transcriptional repression or mRNA degradation (Dweep et al., 2011). miRNAs participate in a wide range of biological processes, including proliferation, differentiation, development, and disease pathogenesis (Li et al., 2011; Bartel, 2004). Notably, numerous studies have demonstrated that miRNAs are abundant in skeletal muscles and exert essential functions in orchestrating gene regulation processes during muscle development (Horak, Novak & Bienertova-Vasku, 2016). Dicer is involved in miRNA maturation, which is greatly reduced by knocking down Dicer expression (Hutvagner et al., 2001). In Dicer knock-out mutants in mice, skeletal muscle development was heavily disrupted (O’Rourke et al., 2007). Additionally, miRNAs affect myogenesis by regulation of myoblast differentiation and proliferation processes (Luo, Nie & Zhang, 2013). For example, miR-133 was implicated in myogenic differentiation through specific modulation of MAML1 and nPTB expression (Luo, Nie & Zhang, 2013). miR-24 was found to affect myoblast differentiation by regulating expression of MEF2D, Myf5, MyoD, Myogenin and MHC (Sun et al., 2008). miR-143 was found to be involved in modulating the proliferation and differentiation of bovine muscle satellite cells by targeting IGFBP5 (Zhang et al., 2017).

To date, miRNAs expressed in skeletal muscle have been identified in some species (e.g., swine (Mai et al., 2016), sheep (Zhao et al., 2016), chicken (Li et al., 2011)), but have not yet been reported in pigeons. Here, we explore miRNA expression profiles in pigeon pectoral muscles over three stages, including 1 day, 28 day and 2 years old, to identify miRNAs and determine their potential roles in pectoral muscle development. Our findings will enhance understanding of the mechanisms underlying rapid development in squabs.

## Materials and methods

### Experimental animals and tissues collection

Thirty-six white king pigeons, including twelve parent pigeons and twenty-four squabs, were obtained from the FengMao pigeon breeding farm (Mianyang, China). The pigeons were housed in individual wire-mesh cages. Pigeons squabs (1-day old) were fed by parents with crop milk. Adult pigeons and 28-day-pigeons were fed with a mixed-grain diet. Water and grit was given ad libtum. Twelve parent pigeons (six males and six females at 2 years old) and twenty-four squabs (mixed sex) were grouped into three age stages (twelve replicates for each stage): the age of 1 day (1d), 28 day (28d) and 2 years old (2yr). The animal experiment was conducted in accordance with protocols approved by the Institutional Animal Care and Use Committee of Sichuan Agricultural University (Permit No. DKY-S20176908). Pigeons were first anesthetized with ether and then euthanized. Subsequently, the bilateral pectorals of each pigeon were collected and weighed. From these tissue samples, samples for small RNA sequencing were immediately frozen in liquid nitrogen and stored at −80 °C until RNA extraction, while samples for hematoxylin and eosin (H&E) staining were fixed in 10% neutral buffered formalin solution.

### Histomorphological examination of pectorals tissue

Pectoral muscle tissues were fixed for 24 h. After being dehydrated in graded alcohol, the specimens were embedded in paraffin and subjected to a microtome (Leica, Wetzlar, Germany). Serial slices at 5 µm thickness were prepared and stained with H&E, and examined by light microscopy.

### RNA isolation and small RNA sequencing

Frozen pectoral muscle samples from nine male pigeons across three age stages (3 pigeons for each stage) were used for total RNA extraction with Invitrogen TRIzol reagent (Thermo Fisher Scientific, Waltham, MA, USA). The integrity and concentration of total RNA samples was assessed with an Agilent 2100 RNA Nano 6000 Assay Kit (Agilent Technologies, Santa Clara, CA, USA). A total RNA sample from each pigeon was individually used for library construction. The library was prepared according to the method and process of Small RNA Sample Preparation Kit (Illumina, RS-200-0048). For each library, small RNAs ranging from 10–45 nt in length were separated from total RNA by polyacrylamide gel electrophoresis and ligated with proprietary adaptors. The modified small RNA molecules were then subjected to RT-PCR. After the library construction was completed, Qubit2.0 was used for preliminary quantitative analysis, and the library was diluted to 1ng/µl. Insert size was assessed using the Agilent Bioanalyzer 2100 system (Agilent Technologies, CA, USA), and after the insert size consistent with expectations, qualified insert size was accurate quantitative using Taqman fluorescence probe of AB Step One Plus Real-Time PCR system (Library valid concentration>2nM). Finally, qualified libraries were sequenced by an Illumina Hiseq 2500 platform (Illumina, Inc.; San Diego, CA, USA) as 50 bp single-end reads.

### Identification and differential expression analysis of miRNAs

MicroRNAs in pigeon pectoral muscles were identified as previously described (Wang et al., 2020). Differential miRNA expression analysis was performed across the different developmental stages by using EdgeR (http://www.omicshare.com/tools). In this study, miRNAs were considered to be differentially expressed only when the value of log2Fold-change was at least 1 or  ≤  −1 and the false discovery rate (FDR) was less than 0.05.

### qPCR

Six randomly selected DE miRNAs were subjected to quantitative RT-PCR. Reverse-transcription and PCR amplifications were respectively performed with Mir-X miRNA First-Strand Synthesis Kit (TaKaRa, Otsu, Japan) and SYBR^®^ Premix Ex Taq™ II (TaKaRa, Dalian, China) on a CFX96 Real-Time PCR Detection System (Bio-Rad, Hercules, CA, USA) according to the manufacturer’s protocols. We used three technical replicates for each sample. Expression levels of selected miRNAs were normalized against U6 snRNA. Relative miRNA levels were calculated using the 2^−ΔΔCt^ method. Information on the primer sequences used is available in Table S1.

### miRNA expression patterns analysis

To obtain knowledge about expression patterns of DE miRNAs in pigeon pectoral muscles during development, DE miRNAs were clustered with STEM software using the log normalize data option, with all other settings at default (Ernst & Bar-Joseph, 2006).

### Target genes prediction and enrichment analysis

The TargetScan algorithm was applied to predict the target genes of DE miRNAs (Garcia et al., 2011). Gene Ontology (GO) and KEGG pathway enrichment analyses for DE miRNA target genes were conducted using the R package ClusterProfiler (Yu et al., 2012) with org.Gg.eg.db (Carlson, 2019).

### Dual-luciferase reporter assay

Fragment of *KLF3* (XM_005501266.3) containing the putative cli-miR-20b-5p binding site (WT) or mutant binding site (MUT) were synthesized by Tsingke (Chengdu, Sichuan, China) and cloned into the SacI and XhoI sites of the pmirGLO plasmid (Promega, Madison, WI, USA) at the 3′ end of the firefly luciferase reporter gene. HeLa cells were seeded in 96-well plates, and recombinant pmirGLO vectors with WT or MUT binding sites were co-transfected into these cells with cli-miR-20b-5p mimics or NC mimics (GenePharma, Shanghai, China) using Lipofectamine 3000 reagent. 48 h after transfection, luciferase assay was performed using the Dual-Luciferase Reporter Assay System kit (Promega) according to the manufacturer’s protocols.

### Statistical analysis

Data are presented as the mean ± standard error of the mean (SEM). Statistical analysis was performed using SPSS v.19.0 (IBM, Armonk, NY, USA). Differences between groups were evaluated using ANOVA or Student’s *t*-test; *p*-values less than 0.05 were considered statistically significant.

## Results

### Phenotypic measurements

**Figure 1:**
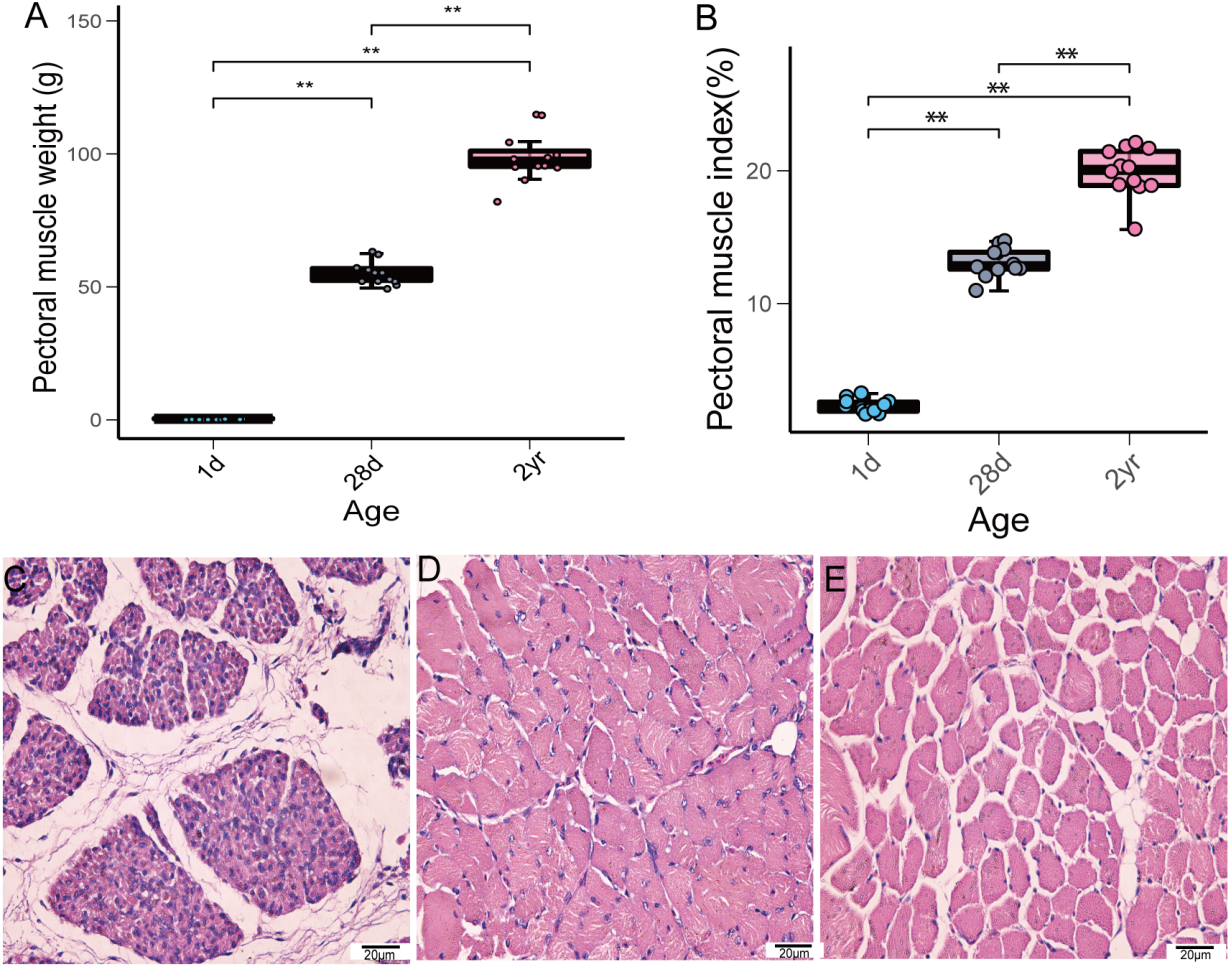
Pectoral muscle weight and H&E staining at the different age stages of pigeons. (A) Pectoral muscle weight (*n* = 12). (B) Pectoral muscle index (*n* = 12). Asterisks (**) denote significant differences at P ≤ 0.01. (C) H&E staining (1d) (400×). (D) H&E staining (28d) (400×). (E) H&E staining (2yr) (400×).

In this study, we investigated the weight of pectoral muscle and histomorphological changes during pigeon development. The weight and index of pectoral muscle gradually increased from the time squabs were 1 day old until the pigeons reached the age of 2 years (Figs. 1A–1B). Furthermore, noticeable histomorphological differences existed in pectoral muscles among the different stages. As depicted in Fig. 1C, the connective tissue between the muscle bundles occupies a large area. From 1 to 28 days of age, the diameter of squab pectoral muscle cells increased gradually and most of the nuclei migrated to the cell membrane edge. The muscle bundles were tightly arranged at 28 days of age (Fig. 1D), while the spacing of muscle bundles conspicuously increased in adult pigeons (Fig. 1E).

### Summary of sequencing data

To identify miRNAs in pectoral muscle during the development of pigeon squabs, we constructed nine small RNA libraries and generated 125.99 million raw reads (Table S2).The average number of raw reads produced for each sample was 14.0 million. High-quality clean reads were obtained after filtering adaptor sequences, contamination and low-quality reads. The proportion of high-quality clean reads ranged from 82.77% to 97.16%. The majority (75.26–93.23%) of the small RNAs in the nine libraries ranged from 21 nt to 24 nt in length (Fig. S1). Of these, the 22-nt category was the most abundant.

### miRNA transcriptome profiles during pectoral muscle development

We identified 591 mature miRNAs corresponding to 396 pre-miRNAs in pigeon pectoral muscle across the three age stages (Table 1). Among these miRNA candidates, 304 known pigeon miRNAs corresponded to 169 known pigeon pre-miRNAs (Table S3). For the conserved pigeon miRNAs, 201 corresponded to 163 pre-miRNAs in two other avian species (*Gallus gallus* and *Taeniopygia guttata*) and mammalian species (Table S4). 64 candidate pre-miRNAs yielded 86 putative novel miRNAs (Table S5).

**Table 1:** Pigeon miRNAs identified in nine sRNA libraries.

Group (number of pre-miRNA/miRNA)	1d	28d	2yr	Total
Pigeon known miRNAs	161/293	145/264	140/246	169/304
Pigeon conserved miRNAs	113/136	84/105	65/81	163/201
Pigeon putative novel miRNAs	39/57	34/48	30/37	64/86

**Figure 2:**
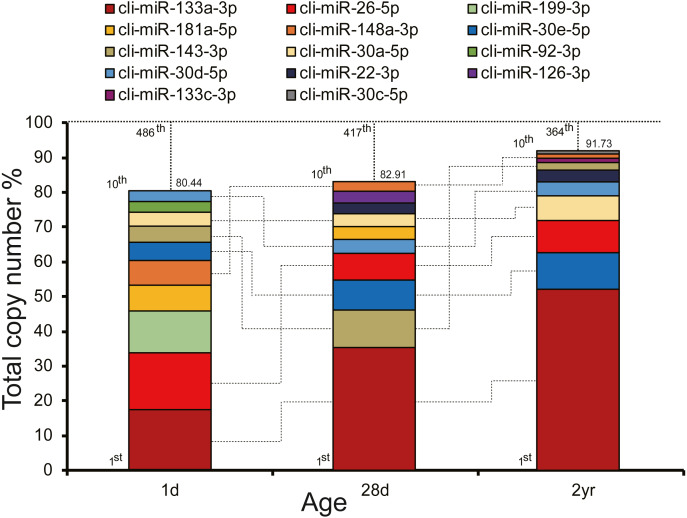
Top 10 highly abundant miRNAs in pigeon muscle at different age stages. The dashed vertical lines denote the cumulative percentage of the top 10 unique miRNAs. Seven miRNAs within the top 10 in each stage are shared across all three stages and are connected by lines.

To unveil potential functions of miRNAs in pigeon pectoral muscle during different developmental stages, we ranked the miRNAs according to their expression abundances. As depicted in Fig. 2, expression abundances of the top 10 unique miRNAs accounted for 80.44%–91.73% of the total counts in each stage. The unified set of the top 10 unique miRNAs over three stages corresponded to 14 kinds of unique miRNAs, seven of which (cli-miR-133a-3p, cli-miR-26-5p, cli-miR-148a-3p, cli-miR-30e-5p, cli-miR-143-3p, cli-miR-30a-5p, and cli-miR-30d-5p) were shared by all stages.

**Figure 3:**
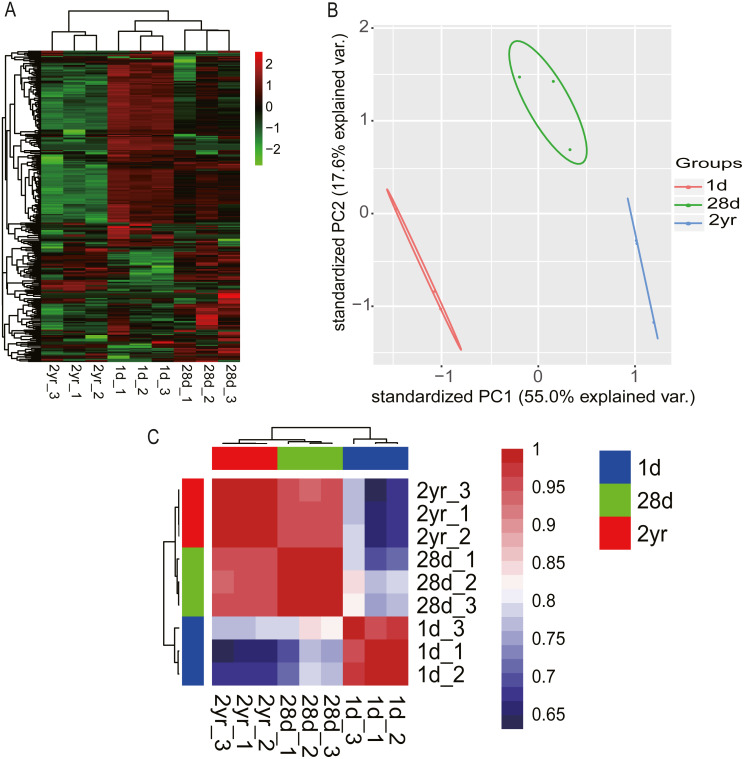
miRNA transcriptome profiles in pigeon pectoral muscle across different age stages. (A) Heat map diagram of HCL analysis for miRNAs among all nine miRNA libraries. (B) PCA of miRNAs across all nine libraries. (C) Heat map matrix of Pearson correlations between all nine libraries.

Hierarchical clustering (HCL) analysis and principal component analysis (PCA) were performed using RPM values of miRNAs in nine miRNA libraries (miRNAs with fewer than 10 counted reads were removed). Results indicated that miRNA expression data in pigeon pectoral muscle clustered into two groups according to developmental stages. The largest cluster was composed of the younger squabs (1d and 28d) and separated from 2yr pigeons (Fig. 3A), suggesting that developmental time contributes to discrepancies among miRNA transcriptomes in pigeon pectoral muscle. These dissimilarities were confirmed by PCA (Fig. 3B) and Pearson’s correlation matrix (Fig. 3C).

### Differential expression analysis

**Figure 4:**
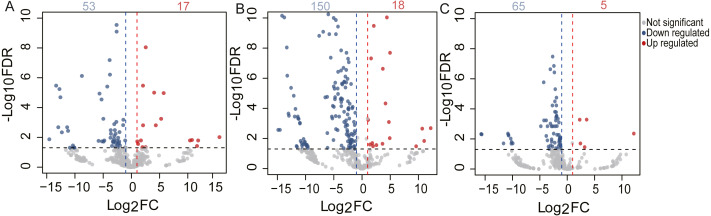
Volcano plot of the DE miRNAs between two different age groups. The *x*-axis indicates the difference in expression level on a log2 (fold change). The *y*-axis represents the corresponding false discovery rate on a negative log10(FDR).

**Figure 5:**
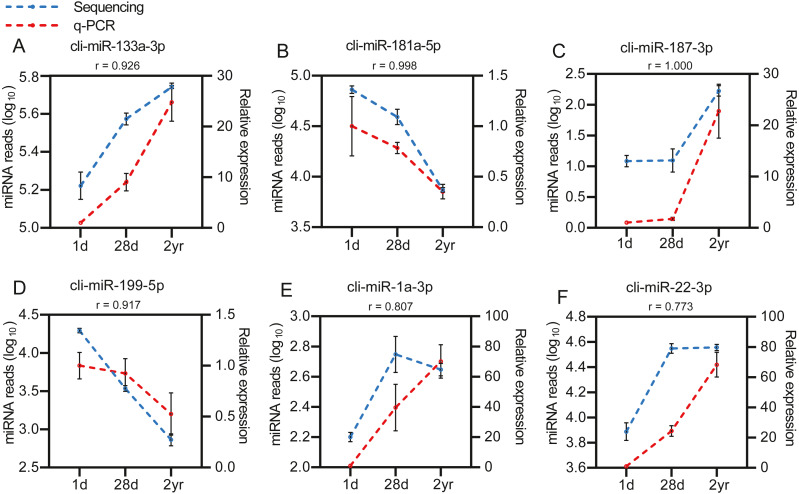
Validation of the sequencing data using qPCR for 6 randomly selected DE miRNAs (*n*= 3). (A) cli-miR-133a-3p (B) cli-miR-181a-5p (C) cli-miR-187-3p (D) cli-miR-199-5p (E) cli-miR-1a-3p (F) cli-miR-22-3p.

To identify the DE miRNAs among different age stages, differential expression analysis was performed by taking | log_2_(fold change)| ≥ 1 and FDR < 0.05 as the cut-off values after removing miRNAs with fewer than 10 counted reads. 189 DE miRNAs were screened out during pigeon development, accounting for 31.98% of total identified miRNAs (Table S6). Notably, three contrasts (1d vs. 28d, 1d vs. 2yr, 28d vs. 2yr) screened out 70, 168, and 70 DE miRNAs, respectively (Fig. 4). To validate the small RNA sequencing results, 6 miRNAs (miR-133a-3p, miR-181a-5p, miR-187-3p, miR-199-5p, miR-1a-3p, miR-22-3p) were randomly selected to perform a qPCR assay. There was good correlation between qPCR and sequencing data (Pearson *r* = 0.904 ± 0.095, *n* = 6, Fig. 5).

**Figure 6:**
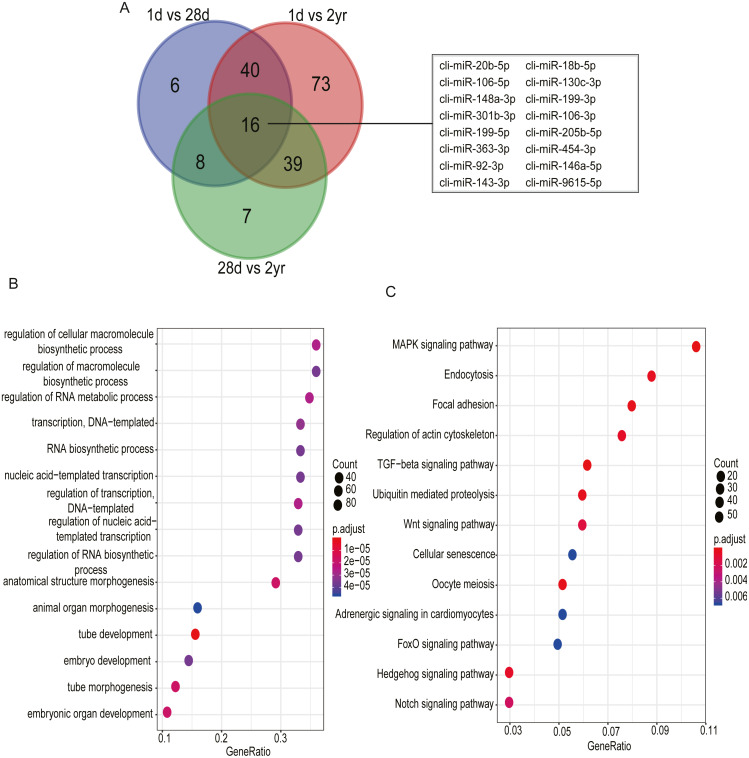
Go and KEGG enrichment analysis for 16 common differentially expressed miRNAs in pectoral muscle during pigeon development. (A) Venn plot revealed that 16 common differentially expressed miRNAs existed in the three different age stages. (B) Gene ontology enrichment analyzed by clusterProfiler. (C) KEGG pathway enrichment analysis. 15 representative enriched Go terms or KEGG pathways are listed. GeneRatio: the ratio of the number of target genes in the GO or KEGG category to that of the annotated genes in the GO or KEGG database.

Among DE miRNAs, there were 16 miRNAs overlapped between the comparisons of 1d vs. 28d, 1d vs. 2yr and 28d vs. 2yr (Fig. 6A). Function analysis showed that the target genes of 16 overlapped DE miRNAs mainly related with regulation of cellular macromolecule biosynthetic process (GO:2000112), regulation of macromolecule biosynthetic process (GO:0010556), regulation of RNA metabolic process (GO:0051252), MAPK signaling pahway and regulation of actin cytoskeleton, etc (Figs. 6B–6C and Table S7).

### DE miRNA expression patterns and functional enrichment analysis

**Figure 7:**
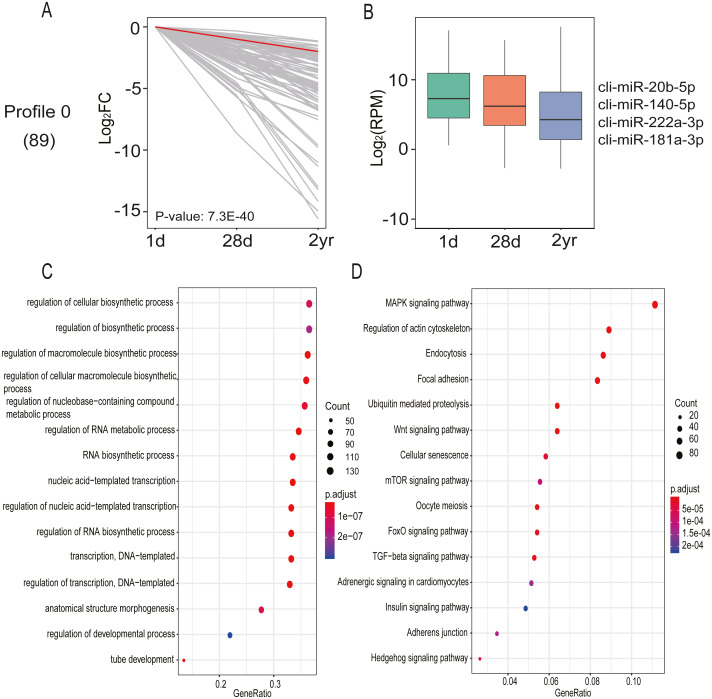
miRNA expression analysis by STEM clustering and functional enrichment analysis. (A) Line plot shows fold changes. One representative miRNA is highlighted in red. (B) Box plot shows expression levels. Representative miRNAs are listed at the right. (C) Gene ontology enrichment analyzed by clusterProfiler. GeneRatio: the ratio of the number of target genes in the GO category to that of the annotated genes in the GO database. (D) KEGG pathway enrichment analysis. 15 representative enriched Go terms orKEGG pathways are listed. GeneRatio: the ratio of the number of target genes in the KEGG category to that of the annotated genes in the KEGG database.

DE miRNA expression patterns in pectoral muscles during pigeon development was assessed with STEM software. A single significant model profile (profile 0) was generated from the 20 distinct expression patterns (Fig. 7A and Table S8). Profile 0 comprised 89 miRNAs and their expression levels progressively declined across all three age stages. It is thought that miRNAs exert their function in a dose-dependent manner (Carlsbecker et al., 2010); hence, only miRNAs with relatively high expression abundance (>1,000 read counts) were employed for potential target gene prediction. As shown in Fig. 7B, the target genes of miRNAs in profile 0 are implicated in the regulation of macromolecule biosynthetic process (GO:0010556), tube development (GO:0035295), anatomical structure morphogenesis (GO:0009653), regulation of cellular biosynthetic process (GO:0031326), and regulation of developmental process (GO:0050793) (Table S9). Additionally, KEGG pathway enrichment analysis found miRNAs in profile 0 are involved in 22 pathways, including MAPK, Wnt, mTOR, TGF-*β*, FoxO and Hedgehog signaling pathways (Fig. 7C and Table S9). These results indicated that these miRNAs carry out an array of vital functions during pectoral muscle development in pigeons.

### Target verification of miR-20b-5p

**Figure 8:**
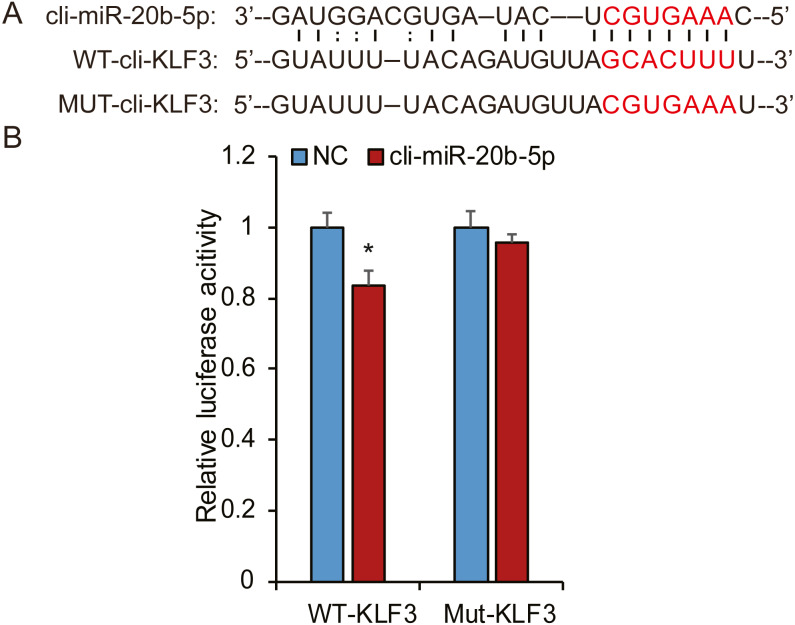
Cli-miR-20b-5p directly targets pigeon KLF3. (A) The predicted binding site and mutated sites of cli-miR-20b-5p in pigeon KLF3. (B) Dual luciferase reporter assay to detect targeting of pigeon KLF3 mRNA by cli-miR-20b-5p in HeLa cells (*n* = 3). The results are shown as mean ±SE. * *p* <0.05.

Based on bioinformatic analysis, we characterized the putative binding sites in the pigeon KLF3 mRNA with miR-20b-5p (Fig. 8A), suggesting this miRNA likely interacted with KLF3 mRNA and repressed its expression. To determine whether miR-20b-5p directly targets the KLF3 mRNA, we performed luciferase assays using a dual-luciferase reporter system containing either wild-type or mutated fragments of KLF3 mRNA. As shown in Fig. 8B, cli-miR-20b-5p conspicuously decreased the relative luciferase activity of wild-type reporter of KLF3 mRNA. In contrast, no notable decrease in activity was observed with the mutant reporters, confirming that miR-20b-5p directly targets KLF3 mRNA.

## Discussion

Pigeon squabs are altrices and have an extraordinarily high growth rate. At 28 days post-hatching, the body weight of pigeons is about 25 times that of newly hatched squabs. In particular, pectoral muscle weight increases by a striking 173.3-fold from 1- to 35-day-old birds (Gao et al., 2016). In the present study, we observed morphological change of the pectoral muscle in pigeons and identified the involved miRNAs and their expression levels at different age stages, including 1d, 28d, and 2yr, representing newly hatched, “weaning” and adult stages, respectively. We identified 591 mature miRNAs in the pectoral muscle during development, and determined differentially expressed miRNAs. Some of these miRNAs have been previously shown to regulate muscle development and regeneration, including miR-133, miR-1, miR-21, miR-499, miR-26, miR-222, and miR-181 (Bai et al., 2015; Luo, Nie & Zhang, 2013). Of these, miR-133 and miR-1 belong to the MyomiRs, which play important roles in controlling muscle myosin content, myofiber identity and muscle performance (Zuo et al., 2015). It was reported that gga-miR-133a-3p could positively regulate myogenic differentiation by inhibiting PRRX1 expression (Guo et al., 2018). Our results indicated that miR-133a-3p exhibited the highest abundance in pigeon pectoral muscle across all age stages. Intriguingly, miR-133a-3p is also the most abundant miRNA in the *longissimus dorsi* muscle of pigs during early developmental stages (prenatal embryonic day 90–30 day), but not in 180-day-old and 7-year-old pigs (Mai et al., 2016). Furthermore, the relative abundance of miR-133a-3p increased gradually from 1d to 2yr. These results indicate that miR-133a-3p might be crucial for the development of pigeon pectoral muscles. In addition to miR-133a-3p, another six miRNAs (cli-miR-26-5p, cli-miR-148a-3p, cli-miR-30e-5p, cli-miR-143-3p, cli-miR-30a-5p, and cli-miR-30d-5p) also ranked within the ten most highly expressed miRNAs in each stage and overlapped in the three age stages. These highly expressed miRNAs are also closely linked with myogenesis and muscle development. One previous study found overexpression of miR-26a promotes myogenesis whereby induction of creatine kinase activity enhanced myoD and myogenin mRNA expression levels (Wong & Tellam, 2008). miR-148a interacted with a myogenesis inhibitor (Rho-associated coiled-coil containing protein kinase 1 [ROCK1] gene), to promote myogenic differentiation (Zhang et al., 2012), whereas miR-30 family miRNAs affected myoblast terminal differentiation by repressing expression of two negative regulators of myogenesis (*Smarcd2* and *Snai2*) (Guess et al., 2015).

In this study, we identified a total of 189 differentially expressed (DE) miRNAs in pigeon pectoral muscle over three age stages. Of these, 89 (e.g., miR-20b-5p, miR-181a-3p) were significantly clustered in a progressively decreasing expression profile. The target genes of DE miRNAs (number of read counts >1000) in this profile were enriched mostly in biosynthesis-related GO categories including ‘regulation of cellular biosynthetic processes’ and ‘regulation of macromolecule biosynthetic processes.’ It has been well established that enhanced protein synthesis and reduced protein turnover result in muscle hypertrophy (Johnson, Smith & Yong Chung, 2014), which our functional enrichment analysis results concur with. IGF-I is a crucial modulator of muscle development, which has a direct anabolic effect on muscle—for instance, increased protein production (Adams, 2000). Our results indicated that some miRNAs with progressively decreasing expression (e.g., cli-miR-19a-3p, cli-miR-130c-3p, cli-miR-99-5p, and cli-miR-15c-5p) were predicted to target pigeon IGF-I or IGF-I receptor mRNA, implying that these DE miRNAs might regulate muscle development and growth by targeting IGF-I and IGF-1R. Furthermore, KEGG pathway enrichment analysis revealed that the same target genes are involved in MAPK, mTOR, Wnt, and TGF-*β* signaling pathways, which have been demonstrated to be directly relevant to skeletal muscle development and growth (Girardi & Le Grand, 2018; Hatfield et al., 2015; Hua et al., 2016; Keren Tamir, & Bengal, 2006). For example, TGF-*β* is a multifunctional regulator that modulates cell proliferation, differentiation, morphogenesis, tissue homeostasis, and regeneration (Massague, 2012). A member of the TGF-*β* family, myostatin, acts as a negative regulator of skeletal muscle growth by inhibiting activation and self-renewal of the satellite cells (McCroskery et al., 2003) Additionally, the p38 MAPK pathway is activated during myogenesis. As a result, activated p38 signaling affects the activities of transcription factors from the MyoD and MEF2 families, promoting expression of muscle-specific genes (Keren Tamir, & Bengal, 2006). Notably, we found that cli-miR-20b-5p may be involved in regulation of MAPK signaling pathways by directly targeting *KLF3*. KLF3, a member of the Krüppel-like factor (KLF) family of transcription factors, is enriched at promoters of many muscle genes including muscle creatine kinase (MCK), myosin heavy chain IIa, and skeletal *α*-actin; it is upregulated during skeletal myocyte differentiation (Himeda et al., 2010). Serum response factor (SRF) is a widely expressed transcription factor activated by the MAPK signaling pathway (Ohrnberger et al., 2015). As a KLF3 interaction partner, SRF exhibits strong synergy with KLF3 in trans-activating the MCK promoter that is expressed upon differentiation of myoblasts into myotubes (Himeda et al., 2010). Taking these previous findings together with our luciferase assay result, we speculate that progressive downregulation of miR-20b-5p potentially alleviates inhibition of *KLF3* expression, which in turn affect muscle development.

## Conclusions

In summary, we identified a total of 304 known miRNAs, 201 conserved miRNAs, and 86 novel miRNAs in pigeon pectoral muscle at different age stages using small RNA sequencing. The cli-miR-133a-3p had the highest abundance in pigeon pectorals across all age stages. DE miRNAs with progressively decreasing expression were mainly involved in the regulation of cellular biosynthetic process and enriched in MAPK and TGF-*β* signaling pathways. Our findings expand the repertoire of pigeon miRNAs and may aid in elucidating the mechanisms of rapid development in squabs.

##  Supplemental Information

10.7717/peerj.11438/supp-1 Supplemental Information 1 Length distributions of small RNAs in nine libraries

10.7717/peerj.11438/supp-2 Supplemental Information 2 Primer sequences of the qPCR assays

10.7717/peerj.11438/supp-3 Supplemental Information 3 Sequencing data overview

10.7717/peerj.11438/supp-4 Supplemental Information 4 Known miRNA group identified in this study

10.7717/peerj.11438/supp-5 Supplemental Information 5 Conserved miRNA group identified in this study

10.7717/peerj.11438/supp-6 Supplemental Information 6 Novel miRNA group identified in this study

10.7717/peerj.11438/supp-7 Supplemental Information 7 List of differentially expressed miRNAs during pectorals development

10.7717/peerj.11438/supp-8 Supplemental Information 8 Function analysis for 16 overlapped DE miRNAs

10.7717/peerj.11438/supp-9 Supplemental Information 9 Stem analysis results

10.7717/peerj.11438/supp-10 Supplemental Information 10 Function analysis for DE miRNAs in profile 0

10.7717/peerj.11438/supp-11 Supplemental Information 11 qPCR raw data

10.7717/peerj.11438/supp-12 Supplemental Information 12 Relative luciferase activity raw data

## Additional Information and Declarations

### Data Availability

The following information was supplied regarding data availability: Data are available at NCBI GEO: GSE153009.
