# Supplementary figures and images for: Identification and expression pattern analysis of miRNAs in pectoral muscle during pigeon (Columba livia) development
Source: PeerJ. 2021 Jun 23;9:e11438. doi: 10.7717/peerj.11438 (PMC8234919; doi:10.7717/peerj.11438)

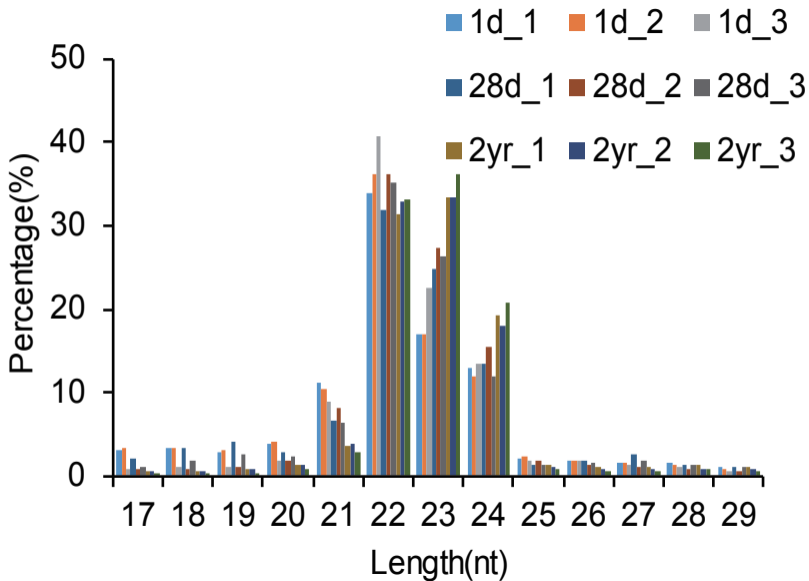

Supplement: Supplemental Information 1 [file peerj-09-11438-s001.pdf]
