# Supplementary material for: Identification and expression pattern analysis of miRNAs in pectoral muscle during pigeon (Columba livia) development
Source: PeerJ. 2021 Jun 23;9:e11438. doi: 10.7717/peerj.11438 (PMC8234919; doi:10.7717/peerj.11438)
Supplement: Supplemental Information 2 [file peerj-09-11438-s002.docx]

**Table S1.** **Primer sequences of the qPCR assays**

| **Name** | **Primer** | **Primer sequence (5' - 3')** |
| --- | --- | --- |
| cli-miR-133a-3p | FW Primer | TTTGGTCCCCTTCAACCAGCTGT |
| cli-miR-181a-5p | FW Primer | AACATTCAACGCTGTCGGTGAGT |
| cli-miR-187-3p | FW Primer | TCGTGTCTTGTGTTGCAGCCAGA |
| cli-miR-199-5p | FW Primer | CCCAGTGTTCAGACTACCTGTTC |
| cli-miR-1a-3p | FW Primer | TGGAATGTAAAGAAGTATGTAT |
| cli-miR-22-3p | FW Primer | AAGCTGCCAGTTGAAGAACTGT |
| U6 | FW Primer | GCAGGGGCCATGCTAATCTTCTCTGTATCG |

Reverse primer was provided by kit manufacturer.
